# Blood Cues Induce Antipredator Behavior in Nile Tilapia Conspecifics

**DOI:** 10.1371/journal.pone.0054642

**Published:** 2013-01-18

**Authors:** Rodrigo Egydio Barreto, Caio Akira Miyai, Fabio Henrique Carretero Sanches, Percília Cardoso Giaquinto, Helton Carlos Delicio, Gilson Luiz Volpato

**Affiliations:** Instituto de Biociências, UNESP – Universidad Estadual Paulista, Campus de Botucatu – Rubião Jr., Departamento de Fisiologia, 18618-970, Botucatu, São Paulo, Brazil; The Australian National University, Australia

## Abstract

In this study, we show that the fish Nile tilapia displays an antipredator response to chemical cues present in the blood of conspecifics. This is the first report of alarm response induced by blood-borne chemical cues in fish. There is a body of evidence showing that chemical cues from epidermal ‘club’ cells elicit an alarm reaction in fish. However, the chemical cues of these ‘club’ cells are restricted to certain species of fish. Thus, as a parsimonious explanation, we assume that an alarm response to blood cues is a generalized response among animals because it occurs in mammals, birds and protostomian animals. Moreover, our results suggest that researchers must use caution when studying chemically induced alarm reactions because it is difficult to separate club cell cues from traces of blood.

## Introduction

Aquatic animals use chemical cues for predator recognition and defensive behavior [1]–[3]. These cues are readily used because they quickly dissolve and spread in the water, and they are especially relevant when visibility is low [4]–[6]. In the context of predator-prey interactions, chemical cues directly or indirectly indicate the presence of a predator, either by the physical presence of a predator odor [4] or by chemical cues from threatened [7], [8] or injured [1] prey. The perception of chemical cues has strong implications for prey survival because it allows prey animals to anticipate a potential predator attack and to employ antipredator responses accordingly [1], [9].

In fish, a common chemical cue comes from injury of a prey animal as a result of a predator attack. These cues have been particularly well studied in species from the superorders Ostariophysi and Acanthopterygii (Perciformes), which have ‘club’ cells in their epidermis that may produce and store these putative chemical alarm cues [1], [10]–[13]. These cues are released into the water by mechanical damage to the skin during the capture stage of a predation event, eliciting alarm reactions on conspecifics, that comprise behavioral and physiological changes [1], [4], [14]–[20].

However, recent evidence suggests that a new approach to investigating antipredator behavior mediated by chemical alarm cues is imperative. In the pintado catfish *Pseudoplatystoma corruscans*, well-fed individuals produce a more easily detectable chemical cue than do food-restricted individuals, regardless of the number of club cells [20]. This finding suggests that the production of the chemical alarm cue is independent of the number of ‘club’ cells. In the fathead minnow *Pimephales promelas*, adult fish respond to extracts from larval skin that still have no visible club cells [21]. In fact, other functions of ‘club’ cells have been observed, such as innate immunological defense [22].

In addition to the ‘club’ cell-based alarm system in fish, other chemical cues could plausibly be passively released as a result of injuries from predator attacks, such as blood-borne chemical cues. In invertebrates, extracts obtained from crushed conspecifics induce an alarm response, e.g., in crustaceans [23], [24], mollusks [25] and echinoids [26]. Extracts of crushed conspecifics always contain hemolymph. Recently, hemolymph from conspecifics was shown to induce an alarm reaction in the spiny lobster *Panulirus argus* [27], the bumblebee *Bombus vosnesenskii* and the honeybee *Apis mellifera* [28]. In terrestrial vertebrates, alarm reactions and avoidance of an area with conspecific blood have been observed in chicks [29] and rats [30], [31]. Conspecific blood also causes olfactory inspection of the surrounding area and stretched locomotion in cattle [32]. In this study, we show that blood-borne chemical cues induce antipredator behavior in conspecific fish. Specifically, fish exposed to diluted fish blood and collected without skin injury decrease locomotion and show an increased latency to feed. As an animal model, we used the cichlid fish Nile tilapia, a species that displays an alarm reaction to chemical cues from conspecific’s injured skin that contains club cells [19].

## Materials and Methods

### Animal Welfare Statement

This research agrees with the Ethical Principles in Animal Research adopted by the National Council for the Control of Animal Experimentation - Brazil (CONCEA - Conselho Nacional de Controle de Experimentacão Animal - Brazil) and was approved by the Ethical Committee for Animal Research from the Instituto de Biociências/UNESP (CEUA - Comissão de Ética no Uso de Animais), protocol 237.

### Fish

Two fish species from hatcheries were used in the experiment: the Nile tilapia *Oreochromis niloticus* (Linnaeus, 1759) and the swordtail (blood red type) *Xyphophorus helleri* (Heckel, 1848). There was no previous contact between these two species. All hatchery-grown Nile tilapia used in the experiment and as blood donors were obtained from the same stock population. The stock population consisted of juvenile Nile tilapia of both sexes with a mean length of 7.6±1.1 cm and a mass of 14.1±5.7 g; the population was maintained in an indoor 2000-L tank (approximately 1 fish/20 l; holding density = approximately 0.7 g/l) for approximately 3 months. The stock tank was supplied with constant aeration and a continuous flow of dechlorinated water. During this time, the temperature averaged 23±1°C, and the water was maintained at low ammonia (<0.25 ppm) and nitrite (<0.50 ppm) levels. The swordtails were only used as blood donors, and they were obtained from a fish dealer 72 h before experimentation. Swordtails were adult individuals of both sexes with a mean body length of 6.0±0.3 cm and a mass of 4.0±0.6 g. They were maintained in a 52.5-L glass aquarium (50×30×35 cm; total water volume = 48 l; approximately 1 fish/2.4 l; holding density = approximately 0.8 g/l). At the fish hatchery, the photoperiod was from 06∶00 to 18∶00 under a light–dark cycle of 12 h light and 12 h dark; this photoperiod was controlled by a timer, with an abrupt transition between light and dark (artificial illumination; daylight fluorescent tube; approximately 350 lx). Food (Nile tilapia: 32% protein, Presence®, Evialis do Brasil Nutrição Animal, Paulínia, SP, Brasil; swordtail: commercial fish flakes, TetraMin Tropical Crisps®) was offered to satiation once per day.

### Experimental Design: The Effects of Conspecific Blood on Nile Tilapia Behavior

The basic strategy of this study was to evaluate locomotion and latency to feed in the Nile tilapia exposed to conspecific blood, a chemical cue hypothesized herein as a chemical that might induce anti-predator reactions in fish. The trials consisted of exposing individual Nile tilapia to one of four chemical cues (10 tilapia for each treatment): (1) Nile tilapia blood (a conspecific fish chemical cue), (2) swordtail blood {an allopatric, unfamiliar, heterospecific chemical cue that served as a control – based on Mathis and Smith [33]}, (3) heparin (the blood anticoagulant control), or (4) distilled water as a control. The trials were conducted between 11∶00 and 14∶00, and the order of testing was randomized.

Prior to the experiment, tilapia from a stock tank were placed in the experimental aquaria (28.0×11.4×19.6 cm) in isolation (1 fish per aquarium) for three consecutive days for acclimation. During this time, fish were fed the same fish chow as in the stock tank at six random times of day so they would be habituated to any potential external interference. We provided food totaling 5% of fish body mass per day, a recommended quantity for Nile tilapia [34]. No pellets were leftover. A tripod and camera were also assembled and placed in front each aquarium for 10 min three times during each acclimation day for habituation.

After the acclimation period, the tilapia’s behavior was recorded for 5 min as a baseline for the locomotion measurements. Next, tilapias were provided with 5 ml of one of the four above-mentioned cues. Twenty seconds later, food was deposited onto the water surface from a tube (1.0 m long and approximately 5 cm in diameter), and tilapia behavior was recorded for an additional 5 min.

The weight and standard length of the tilapia were not significantly different between the four treatment groups. The weights of the four groups (g; mean ± SD) were 14.1±6.1 for the conspecific group, 14.4±5.1 for the heterospecific control, 14.2±5.7 for the heparin control and 14.5±5.8 distilled water control (one-way ANOVA; F_(3;36)_ = 0.013; P = 0.998; n = 10). The lengths (cm; mean ± SD) of the groups were 7.6±1.0 for the conspecific group, 7.5±0.9 for the heterospecific control, 7.6±1.0 for the heparin control and 7.7±0.9 for the distilled water control (one-way ANOVA; F_(3;36)_ = 0.025; P = 0.994; n = 10).

### The Test Tank

Each observation aquarium contained a gravel substrate (1 cm high) and an air stone connected to an air pump via plastic tubing. Opaque partitions were placed outside the back and sides of each experimental aquarium to prevent fish from seeing each other or the investigator during the test.

### The Chemical Cue

The chemical cue was deposited onto the water surface of the test-fish aquarium via a syringe attached to plastic tubing affixed to a steel rod and operated from a position 1.0 m from the aquaria. The chemical cue reached each aquarium above the air bubbles emitted from the air stone, which facilitated dispersion. A dye test with methylene blue showed that the colorant was completely distributed throughout the aquarium in less than 15 s.

For each trial, chemical stimuli were prepared from a pool of 20 Nile tilapias or swordtails. To prevent any skin damage to the tilapias and thus avoid contamination of the blood with the putative conspecific alarm cues from the club cells [19], tilapia blood was carefully collected from the gill blood vessels. Approximately 50 µl of blood was collected from the gill blood vessels using a heparinized syringe. To collect blood from the swordtail, we used an insulin syringe (1 ml) attached to a 0.55×20 mm hypodermic needle. Approximately 20–30 µl of blood was collected by cardiac puncture in the swordtails. The pool of blood was deposited into a glass tube and gently swirled for approximately 10 s. A sample of 250 µl was separated with the aid of a micropipette, transferred to a glass beaker, and diluted in 250 ml of chilled distilled water. Aliquots of 5 ml of diluted blood were drawn into a 20-ml syringe and used in the tests. The remaining 15 ml of air inside the syringe ensured that the blood was fully expelled into the test aquarium.

To heparinize the syringe, we drew 1 ml of heparin into a sterile 1-ml insulin sterile syringe and expelled it back into the heparin container. A very small quantity of heparin remained in the needle. The amount inside the needle was removed by drawing and expelling air into the syringe several times. Thus, the actual amount of heparin remaining in the needle was almost negligible. We estimated the amount of heparin per needle by considering the needle as a cylinder: each 0.55×20-mm needle can contain approximately 4.749 µl (mm^3^) of heparin. Thus, each 1 ml of pooled blood potentially contained 94.985 µl of heparin (20 syringes with needles ×4.749 µl of heparin in each needle). Therefore, a sample of 250 µl from the 1-ml pool of blood contained approximately 23.75 µl of heparin. To control for the effect of the heparin that was used as an anti-coagulant, one of our experimental treatments was a 5-ml aliquot from a solution of 190 µl of heparin diluted in 2 l of chilled distilled water ( = 23.75 µl of heparin/250 ml of water, as for the blood stimuli).

### Behavioral Quantification and Data Analyses

Locomotion and latency to feed in Nile tilapia that were exposed to chemical cues were evaluated as indicators of antipredator responses. Locomotion is strongly affected by threatening chemical cues [1], [15], [19]. The response to the chemical cue also involves a change in the priority of stimuli responses (stimulus filtering), as evidenced by the decreased incidence of feeding activity in the presence of an alarm substance [35]. Behavior was quantified from videotape analysis and the observer did not know which treatment the fish was submitted.

The rear wall of each aquarium was marked with vertical (18 cm high from the bottom to the water level) and horizontal lines that bisected the area {adapted from [13]}. Locomotion was measured by recording the number of line crossings of the rear wall before and after injection of chemical stimuli into the test aquarium. A line cross was recorded when most (approximately 75%) of fish’s body crossed the line. A previous study showed that Nile tilapia tend to decrease activity in response to skin-injured alarm chemical stimulus rather than darting back and forth [19]. A darting movement may not result in line crosses, as observed in the common bully *Gobiomorphus cotidianus* [13]. However, in the present study, no darting was observed. For statistical analyses, locomotion values were considered as the deviation from the initial condition (post-stimulus minus baseline). Latency to feed was the time elapsed between depositing food onto the water surface and the fish’s first snatch at a pellet.

The normality and homoscedasticity of the data were evaluated using the Kolmogorov and Smirnov (KS) test and Bartlett's test, respectively. The KS test showed that the data were sampled from populations that follow normal distributions, and Bartlett's test indicated that the differences among the SDs were statistically indistinguishable. Because the data passed for normality and homoscedasticity, we applied a one-way ANOVA complemented by a Student-Newman-Keuls test to analyze the data. Significant differences were considered at α = 0.05.

## Results

**Figure 1:**
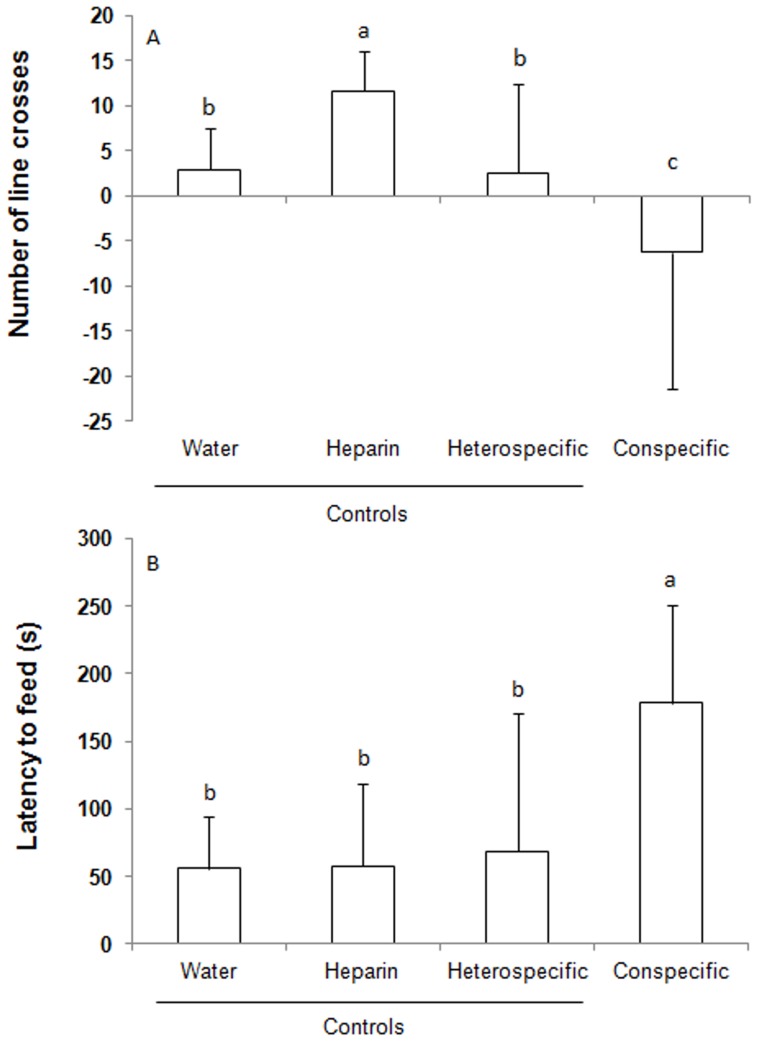
Behavioral response to blood-borne chemical cues in Nile tilapia *Oreochromis niloticus*. The values (mean ± SD; A – number of line crosses and B – latency to feed) that do not share the same letter are significantly different (P<0.05; n = 10; one-way ANOVA followed by a Student-Newman-Keuls test). For the number of line crosses, the values are the deviation from the baseline values. We quantified this variable for 5 min before the treatment to establish a baseline measurement of locomotion, and we quantified locomotion for an additional 5 min after exposure to a chemical stimulus.

The number of line crosses was statistically lower for the group exposed to conspecific blood than it was for the other groups (one-way ANOVA; F_(3;36)_ = 17.501; P<0.0001; Fig. 1a). Fish exposed to heparin displayed increased locomotion in comparison with the other conditions. Fish exposed to distilled water (vehicle control) and heterospecific blood had statistically equal numbers of line crosses. In the case of the latency to snatch the food pellets, fish exposed to conspecific blood delayed feeding in comparison with the other conditions, which were statistically similar to each other (one-way ANOVA; F_(3;36)_ = 6.250; P = 0.0016; Fig. 1b).

## Discussion

Here, we show that chemical cues present in blood induce alarm responses in a fish species. The cichlid Nile tilapia, when exposed to conspecific blood, decreases locomotion and changes the priority of its response to a relevant environmental stimulus (the latency to feed increases). We assume that this response is due to a specific reaction to a chemical alarm cue rather than a general response to a new scent in the water because Nile tilapia do not respond to heterospecific blood.

Methodologically, the primary focus was to eliminate the potential interference of any alarm substance that originated from epidermis ‘club’ cells. A body of evidence suggests that bony fishes from the superorders Ostariophysi and Acanthopterygii (Perciformes) have ‘club’ cells in their epidermis that produce and store a putative chemical alarm cue that is released when the skin is injured, as observed during a predator strike [1], [10]–[13], [19] or if the skin is perforated when blood is collected. Here, we avoided blood contamination with ‘club’ cells chemical cues by carefully collecting blood from the gill vessels, a region where club cells are not present. Therefore, our results represent responses to blood chemical cues from conspecifics.

Freezing and/or decreased activity have been observed in Nile tilapia as a response to skin extracts {the putative alarm cue came from ‘club’ cells [1], [15], [19], [36], [37]. Fish may also change the priority of their response to relevant environmental stimuli, which is considered a defensive response to skin extract. For instance, this cue decreases foraging activity of the convict cichlids *Archocentrus nigrofasciatus* when risk increases [35]. Accordingly, Nile tilapia decrease swimming and stop defending a territory against a conspecific intruder when exposed to skin extract [19]. Nile tilapia also changed their behavior in the same way when exposed to the blood chemical cue, indicating that this behavior is a typical alarm reaction.

We observed that Nile tilapia increased their activity when exposed to heparin. This response was unexpected, and its meaning is difficult to interpret. Fish mucus induces defensive responses in prey invertebrates, and heparin induces similar effects [38]–[40]. However, heparin does not occur naturally in fish mucus but it is structurally similar to chondroitin sulfate A [39], a compound that is present in fish mucus [41], which in turn is associated with the above-mentioned defensive response in prey invertebrates [39]. Thus, in the present study, the response to heparin may be a defensive response, although heparin only increased the swimming activity and did not interfere with the feeding response. This issue must be investigated in future studies. This observation, however, does not invalidate our conclusion that conspecific blood chemical cues induce a fish response that is a typical alarm reaction, even though the blood cues contained heparin as an anticoagulant.

Although there is a body of evidence documenting that the alarm cue that originates from ‘club’ cells can induce adaptive responses, this phenomenon seems more complex. The evolution of these cells is interesting because the fish that signals (releases the alarm cue) must be damaged, and furthermore, most of these fish die, which would not represent any direct fitness advantage. An explanation for the evolution of ‘club ‘cells had been kin selection [42], which suggests that senders increase their inclusive fitness by warning relatives. Nowaday, the explanation relies on the fact that the evolution of a ‘club’ cell-based alarm system is a secondary trait, because these cells have been shown to have other primary adaptive functions. In this line, alarm substance cells have immune functions and provide protection against UV radiation in fish [22], [43]. Thus, evolution of chemical released from club cell is better explained in terms of success of conspecifics that detect and respond adaptively to public predation information [22], [43]. Thus, the club cell system has clear adaptive benefits to the sender and to the receiver, one related to immune system and the other the predator-prey interaction. In the case of a blood-borne chemical alarm cue system, on the other hand, only the receiver needed an adaptive advantage to this trait to be selected and maintained among the individuals. The receiver animal that recognizes a blood cue as a threat and thus displays a defensive behavior increases their probability of surviving. This explanation is consistent with the predictions of the ‘use of public information hypothesis’ regarding an alarm response to hemolymph, as observed in bumblebees and honeybees [28].

Based on the above statements, we conclude that the behavioral change induced by chemical cues present in the blood is a genuine alarm response in fish. This is the first report of this nature in fish. Because an alarm response to blood has also been documented in mammals [30]–[32], birds [29] and some invertebrates [27], [28], we suggest that this blood-borne chemical alarm cue system could be widespread in animals. Moreover, because chemical cues from ‘club’ cells are restricted to certain species, it seems a parsimonious explanation to assume that the alarm reaction elicited by ‘club’ cell chemical cues is a specialized response while the same response elicited by blood cues is widespread among animals (and is most likely an ancient response because it occurs in protostomian animals). Moreover, our results suggest applying additional caution when studying alarm reactions induced by chemical cues because it is difficult to separate club cell cues from traces of blood.
